# Supplementary material for: Spermatocytes have the capacity to segregate chromosomes despite centriole duplication failure
Source: EMBO Rep. 2024 Jun 28;25(8):14. doi: 10.1038/s44319-024-00187-6 (PMC11316026; doi:10.1038/s44319-024-00187-6)
Supplement: Supplementary file 1 — Appendix [file 44319_2024_187_MOESM1_ESM.pdf]

Table of contents:

Appendix Table S1: Genotyping primers used in this study……………………………………………...2
Appendix Table S2: Antibodies used in this study
Primary antibodies……………………………………………………………………………3
Secondary antibodies………………………………………………………………………..4
Appendix Figure S1: Assessment of microtubule-associated factors during meiosis I………..……5-8

Appendix Table S1: Genotyping primers used in this study

Genotyping Primers

Gene	Forward Primer (5"-.....-3')	Reverse Primer (5"-.....-3')
Plk4^{WT}	TCTTGAGGGGAATTAGATAGCA	TCACAGCTATTCCTCACTCAGC
Plk4^{flox}	GCCAGCCCCTCTAATCAGTC	TCACAGCTATTCCTCACTCAGC
Sas4^{WT}	GGGGAGCAGACTTCAACACT	GCTGACACCAAGTGGGAAAT
Sas4^{flox}	TGCTTGCTTGTCTCTCCTGA	GCTGACACCAAGTGGGAAAT
Plk1^{WT}*, Plk1*^{flox}*, and Plk1*^{del}	AGTATGGCAGGCAGCATCTC	ATGCTCCATGGAAAGTCAGG
mCherry-Plk4 Transgene	AGGACGGCGAGTTCATCTAC	TGGTGTAGTCCTCGTTGTGG
mCherry-Plk4 Internal Control	CAAATGTTGCTTGTCTGGTG	GTCAGTCGAGTGCACAGTTT
Cre Transgene	CCATCTGCCACCAGCCAG	TCGCCATCTTCCAGCAGG
Cre Internal Control	ACTGGGATCTTCGAACTCTTTGGAC	GATGTTGGGGCACTGCTCATTCACC
Gdf9 Cre transgene	TCTGATGAAGTCAGGAAGAACC	GAGATGTCCTTCACTCTGATTC

Appendix Table S2: Antibodies used in this study

Primary Antibodies

Antibody	Host	Source	Cat. Number	IF Dilution	IHC
alpha-tubulin	Mouse	Sigma-Aldrich	T9026	1:1000	
alpha-tubulin 4a	Goat	LS Bio	LS-C204216	1:100	
ARID2	Rabbit	Abclonal	A8601	1:100	
AURKA	Rabbit	Protein Tech Group	10297-1-AP	1:50	
AURKB	Rabbit	Abclonal	A19539	1:200	
beta-Catenin (12F7)	Mouse	Santa Cruz Biotech	sc-59737	1:100	
CAMSAP1	Rabbit	Abclonal	A17839	1:100	
CAMSAP2	Rabbit	Protein Tech Group	17880-1-AP	1:200	
CAMSAP3	Mouse	Dr. Masatoshi Takeichi	NA	1:200	
CCDC13	Rabbit	Sigma Life Science	HPA047429	1:500	
CDK5RAP2	Rabbit	Bethyl	IHC-00063	1:250	
CEP192	Goat	Dr. Andrew Holland	NA	1:1000	1:1000
CEP164	Rabbit	Millipore Sigma	ABE2621	1:1000	
Centrin	Rabbit	Dr. Andrew Holland	NA	1:500	
Centrobin	Rabbit	Abclonal	A8277	1:200	
CETN3	Mouse	Abnova	H00001070-M01	1:200	
ch-TOG (CKAP5)	Rabbit	Sigma-Aldrich	HPA040375	1:200	
CLASP1	Rabbit	Abclonal	A7081	1:100	
Cleaved Caspase-3	Rabbit	Cell Signaling Technology	9664 (5A1E)		1:400
delta-tubulin	Rabbit	Abcam	ab214216	1:300	
Dynein	Mouse	Genetex	GTX80684	1:300	
Dynein (DYNALI1)	Mouse	Abnova	H00007802-B01P	1:200	
Dynein (DYNLL1)	Rabbit	Abclonal	A14496	1:100	
gamma-H2AX	Mouse	Thermo Scientific	MA1-2022	1:500	
gamma-tubulin	Mouse	Sigma-Aldrich	T6557	1:1000	1:500
GCP2	Rabbit	Genetex	GTX102281	1:200	
GCP4	Rabbit	Genetex	GTX115949	1:100	
HAUS8	Rabbit	Abclonal	A7847	1:200	
HURP (DLGAP5)	Rabbit	Abclonal	A13575	1:200	
KATNA1	Rabbit	Sigma-Aldrich	HPA036207	1:100	
KATNAL1	Rabbit	Sigma-Aldrich	ABE238	1:100	
KATNB1	Rabbit	Sigma-Aldrich	HPA036207	1:100	
KIF2A	Rabbit	Abclonal	A16392	1:200	
KIF5B	Rabbit	Abclonal	A15284	1:100	
KIF11 (Eg5)	Rabbit	Thomas Scientific	HPA010568	1:300	
KIF18a	Rabbit	Abclonal	A14939	1:200	
KIFC1 (HSET)	Rabbit	Abclonal	A0077	1:100	
mCherry	Mouse	Biolegend	677701	1:500	
mCherry	Rabbit	Thermo Scientific	PA5-34974		1:500
MLH1	Mouse	BD Biosciences	51-1327	1:100	
NEDD1	Mouse	Abcam	ab57336	1:1000	
NuMA	Rabbit	Abcam	ab109262	1:100	
OFD-1	Rabbit	Abclonal	A17567	1:200	
PCNT	Rabbit	Abcam	ab4448	1:200	
PLK1	Rabbit	Genetex	GTX104302	1:200	
PLK4-01 AF-555 conjugate	Rabbit	Dr. Andrew Holland	NA	1:300	1:300
PNA-AF488 conjugate	NA	Molecular Probes (Life Tech)	L21409	1:500	1:500
SYCP1	Rabbit	Thermo Scientific	PA1-16763	1:100	
SYCP3	Mouse	Santa Cruz Biotech	sc-74569	1:50	1:300
SYCP3	Rabbit	Novus Biologicals	NB300-231	1:1000	
SYCP3	Goat	Novus Biologicals	AF3750	1:100	
TACC3	Rabbit	Novus Biologicals	NBP2-67671	1:250	
TRAF3IP1	Rabbit	Abclonal	A8997	1:200	
TPX2	Rabbit	Abclonal	A18327	1:100	
USP24	Rabbit	Abclonal	A0621	1:100	

Secondary Antibodies

Antibody	Host	Source	Cat. Number	IF Dilution	IHC
Goat IgG (H+L) Alexa Fluor 488	Donkey	Invitrogen	A-11055	1:500	1:3000
Goat IgG (H+L) Alexa Fluor 568	Donkey	Invitrogen	A-11057	1:500	1:3000
Goat IgG (H+L) Alexa Fluor 633	Donkey	Invitrogen	A-21082	1:500	1:3000
Rabbit IgG (H+L) Alexa Fluor 488	Donkey	Invitrogen	A-110018	1:500	1:3000
Rabbit IgG (H+L) Alexa Fluor 568	Donkey	Invitrogen	A-11011	1:500	1:3000
Rabbit IgG (H+L) Alexa Fluor 647	Donkey	Invitrogen	A-31573	1:500	1:3000
Mouse IgG (H+L) Alexa Fluor 488	Donkey	Invitrogen	A-11001	1:500	1:3000
Mouse IgG (H+L) Alexa Fluor 568	Donkey	Invitrogen	A-11031	1:500	1:3000
Mouse IgG (H+L) Alexa Fluor 647	Donkey	Invitrogen	A-31571	1:500	1:3000

Appendix Figure S1: Assessment of microtubule associated factors during meiosis I

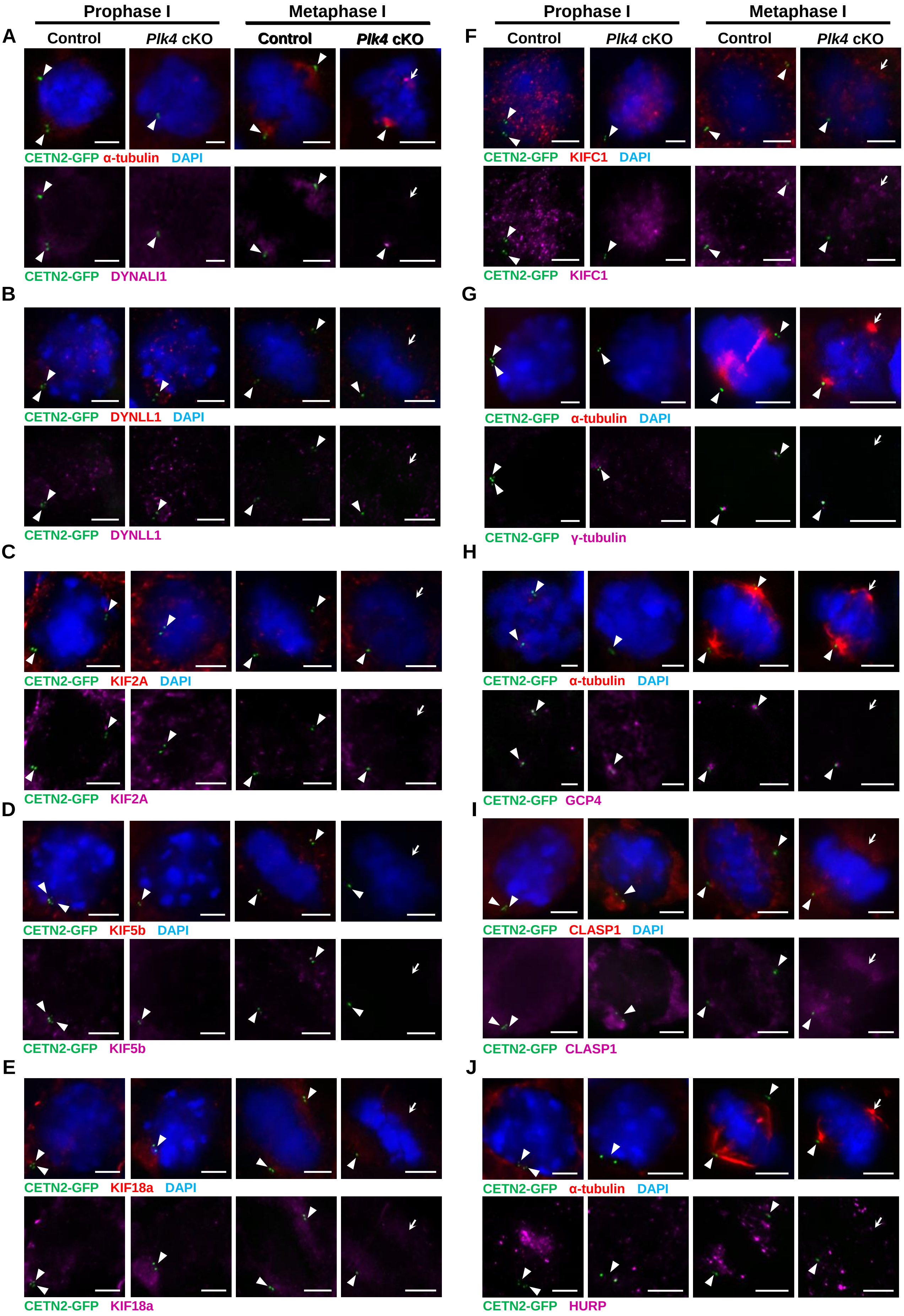

Appendix Figure S1: Assessment of microtubule associated factors during meiosis I

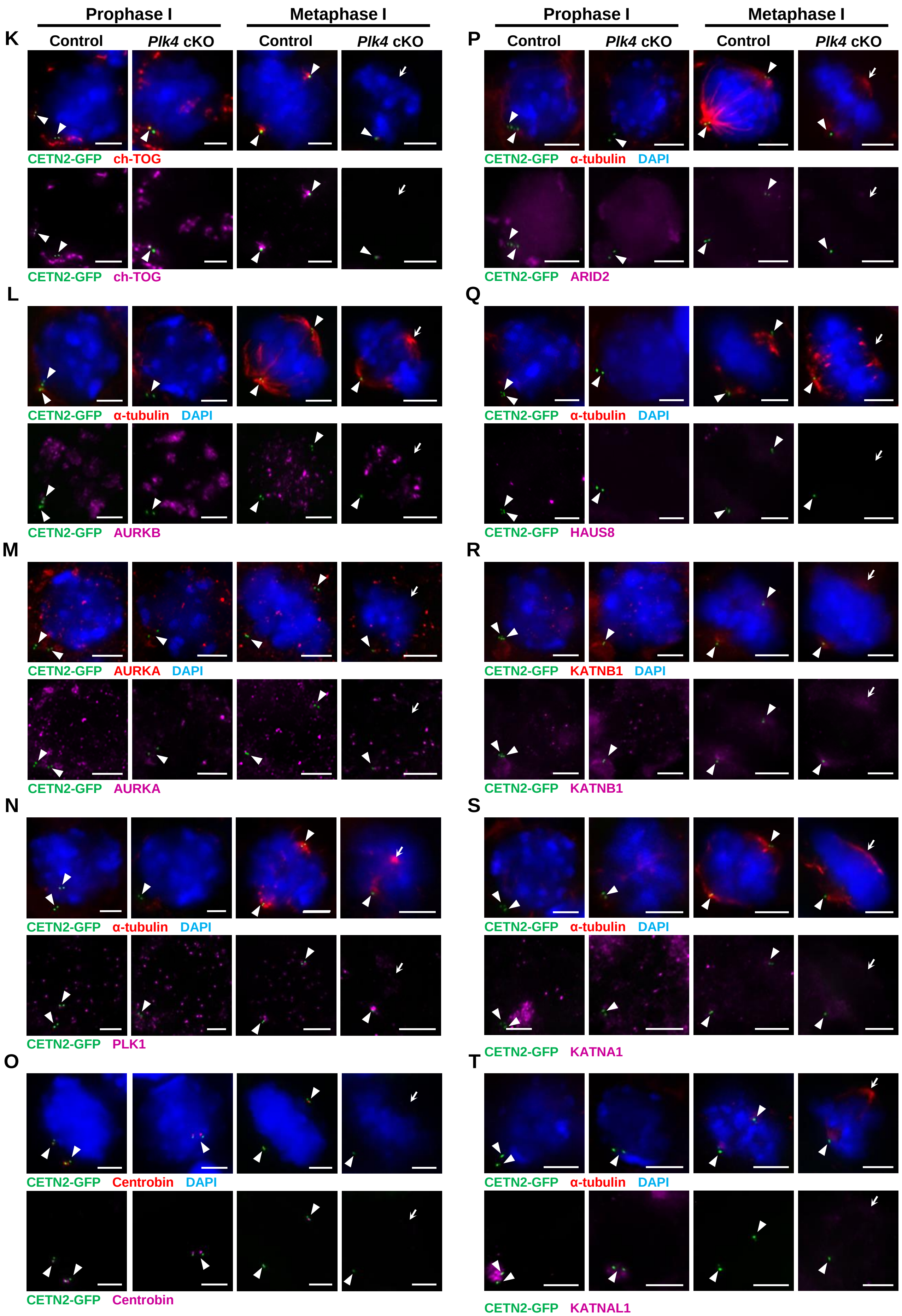

Appendix Figure S1: Assessment of microtubule associated factors during meiosis I

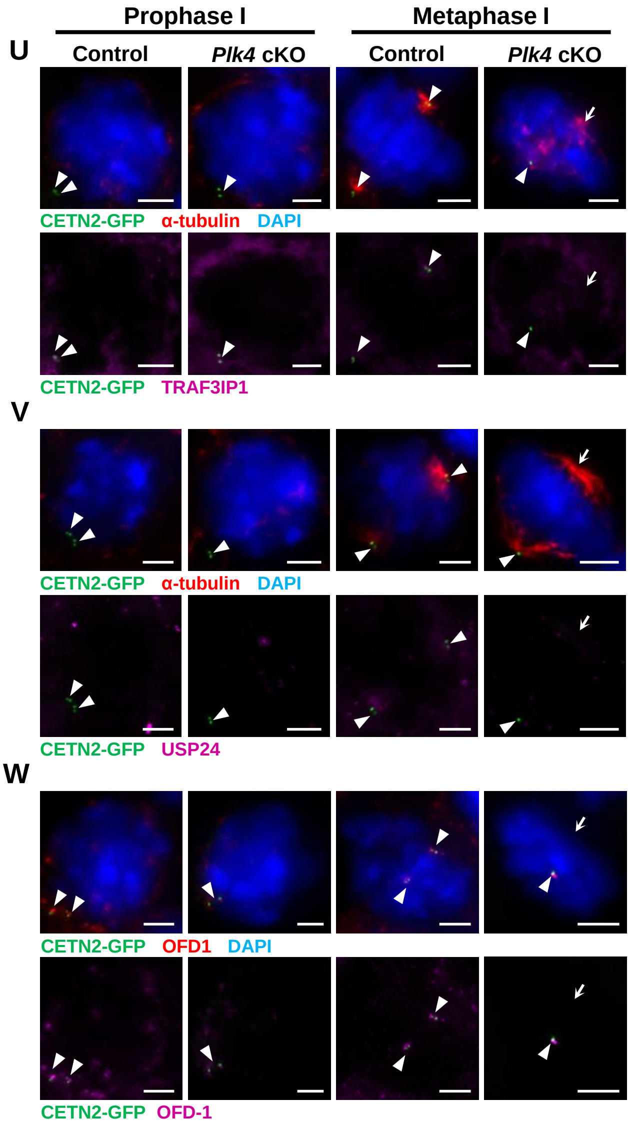

Figure EV4. Assessment of microtubule-associated factors during meiosis I.
Representative image of prophase I (**A-W**) and metaphase I (**A-W**) control and *Plk4* cKO spermatocytes from 24-27 dpp mice expressing CETN2-GFP (green) and stained with DAPI (blue). The white arrowheads indicate the centrosome. The white arrows indicate. Scale bars = 5 µm.

A. Immunolabeled against α-tubulin (red) and DYNALI1 (purple).
B. Immunolabeled against DYNLL1 (red and purple).
C. Immunolabeled against KIF2A (red and purple).
D. Immunolabeled against KIF5b (red and purple).
E. Immunolabeled against KIF18a (red and purple).
F. Immunolabeled against KIFC1 (red and purple).
G. Immunolabeled against α-tubulin (red) and γ-tubulin (purple).
H. Immunolabeled against α-tubulin (red) and GCP4 (purple).
I. Immunolabeled against CLASP1 (red and purple).
J. Immunolabeled against α-tubulin (red) and HURP (purple).
K. Immunolabeled against ch-TOG (red and purple).
L. Immunolabeled against α-tubulin (red) and AURKB (purple).
M. Immunolabeled against AURKA (red and purple).
N. Immunolabeled against α-tubulin (red) and PLK1 (purple).
O. Immunolabeled against centrobin (red and purple).
P. Immunolabeled against α-tubulin (red) and ARID2 (purple).
Q. Immunolabeled against α-tubulin (red) and HAUS8 (purple).
R. Immunolabeled against KATNB1 (red and purple).
S. Immunolabeled against α-tubulin (red) and KATNA1 (purple).
T. Immunolabeled against α-tubulin (red) and KATNAL1 (purple).
U. Immunolabeled against α-tubulin (red) and TRAF3IP1 (purple).
V. Immunolabeled against α-tubulin (red) and USP24 (purple).
W. Immunolabeled against OFD1 (red and purple).
