# Supplementary material for: Spermatocytes have the capacity to segregate chromosomes despite centriole duplication failure
Source: EMBO Rep. 2024 Jun 28;25(8):14. doi: 10.1038/s44319-024-00187-6 (PMC11316026; doi:10.1038/s44319-024-00187-6)
Supplement: Supplementary file 3 — Expanded View Figures [file 44319_2024_187_MOESM3_ESM.pdf]

Expanded View Figures

Figure EV1. Assessment of apoptosis during spermatocytes.

(**A–D**) Cryosections (16 µm thick) of control and *Plk4* cKO mouse testis at 15 (**A**), 19 (**B**), 23 (**C**), and 27 (**D**) dpp, immunolabeled against SYCP3 (green), caspase-3 (red) and stained with DAPI (blue). Zoomed images of individual tubules are shown directly below the corresponding images. White arrows indicate an example of an apoptotic spermatocyte. Scale bar = 100 µm. (**E**) The *Plk1* cKO (*Plk1 flox/flox, Spo11-Cre tg/0*) mouse model has been reported previously to exhibit increased levels of apoptosis in primary spermatocytes and was used as a positive control (Wellard et al, 2021). Cryosections (16 µm thick) of control and *Plk1* cKO (34 dpp) mouse testis immunolabeled against SYCP3 (green), caspase-3 (red) and stained with DAPI (blue). Zoomed images of individual tubules are shown directly below the corresponding images. White arrows indicate an example of an apoptotic spermatocyte. Scale bar = 100 µm. (**F**) Quantification of apoptotic spermatocytes in control, *Plk4* cKO and *Plk1* cKO spermatocytes. Quantification was performed in ≥26 tubules. Error bars show mean ± SEM. *P* values were obtained from two-tailed Student's *t* test. n.s. (not significant) and ****$P < 0.0001$. *P* value = <0.0001.

▶

This is a U.S. Government work and not under copyright protection in the US; foreign copyright protection may apply

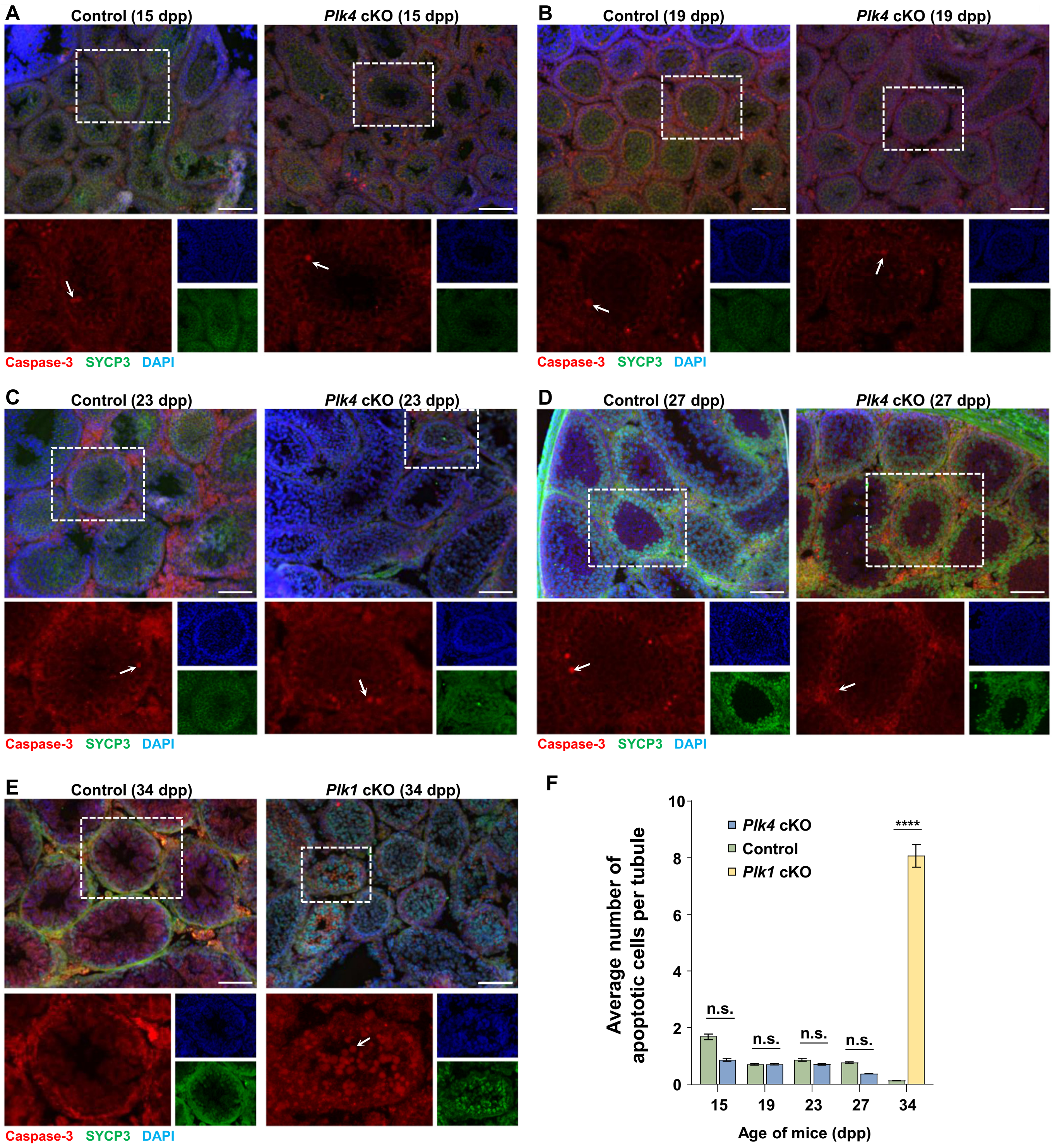

 This is a U.S. Government work and not under copyright protection in the US; foreign copyright protection may apply

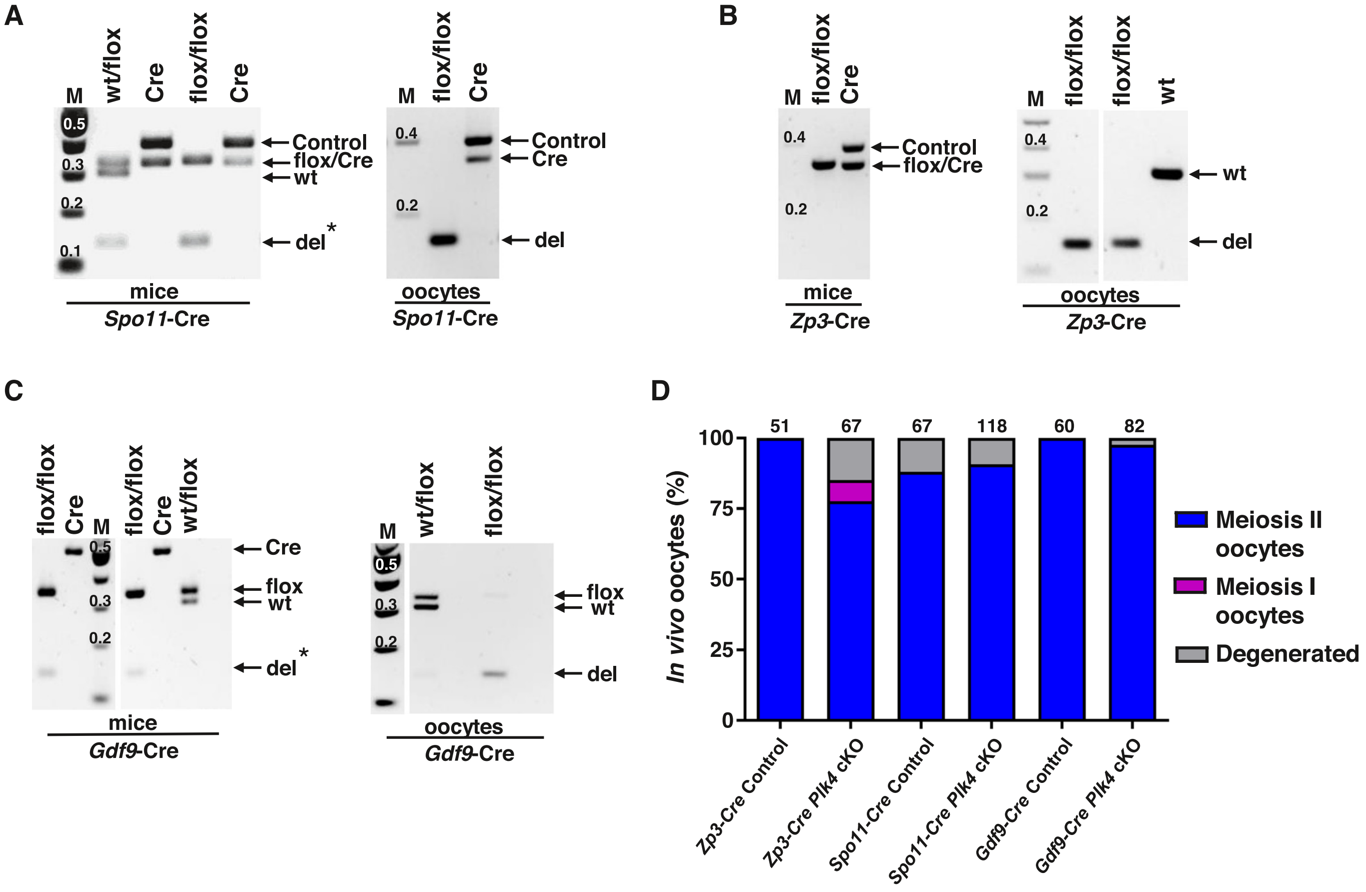

Figure EV2. Assessment of *Plk4* cKO female mice.

(**A–C**) PCR analysis of mouse and oocyte DNA showing the absence of the *flox* allele and presence of the del allele in oocytes after the expression of Cre recombinase driven by the *Spo11* (**A**), *Zp3* (**B**), and *Gdf9* (**C**) promoters. WT *Plk4* band 307 bp, flox floxed *Plk4* band 341 bp, del deletion band 143 bp, M DNA marker, kb kilobases, Cre Cre recombinase band 355 bp for *Spo11*-Cre and *Zp3*-Cre, and 500 bp for *Gdf9*-Cre, Cont internal control band for *Spo11*-Cre and *Zp3*-Cre recombinases 420 bp. *Non-tissue-specific Cre recombinase expression. (**D**) Oocytes from all *Plk4* cKO mice successfully progressed to MII stage in vivo. Mouse age 45-77 days. Oocytes from 3 superovulated mice were analyzed for each genotype. The total number of oocytes quantified from *Zp3*-cre control, *Zp3*-cre *Plk4* cKO, *Spo11*-cre control, *Spo11*-cre *Plk4* cKO, *Gdf9*-cre control and *Gdf9*-cre *Plk4* cKO mice were 51, 67, 67, 118, 60, and 82, respectively.

This is a U.S. Government work and not under copyright protection in the US; foreign copyright protection may apply

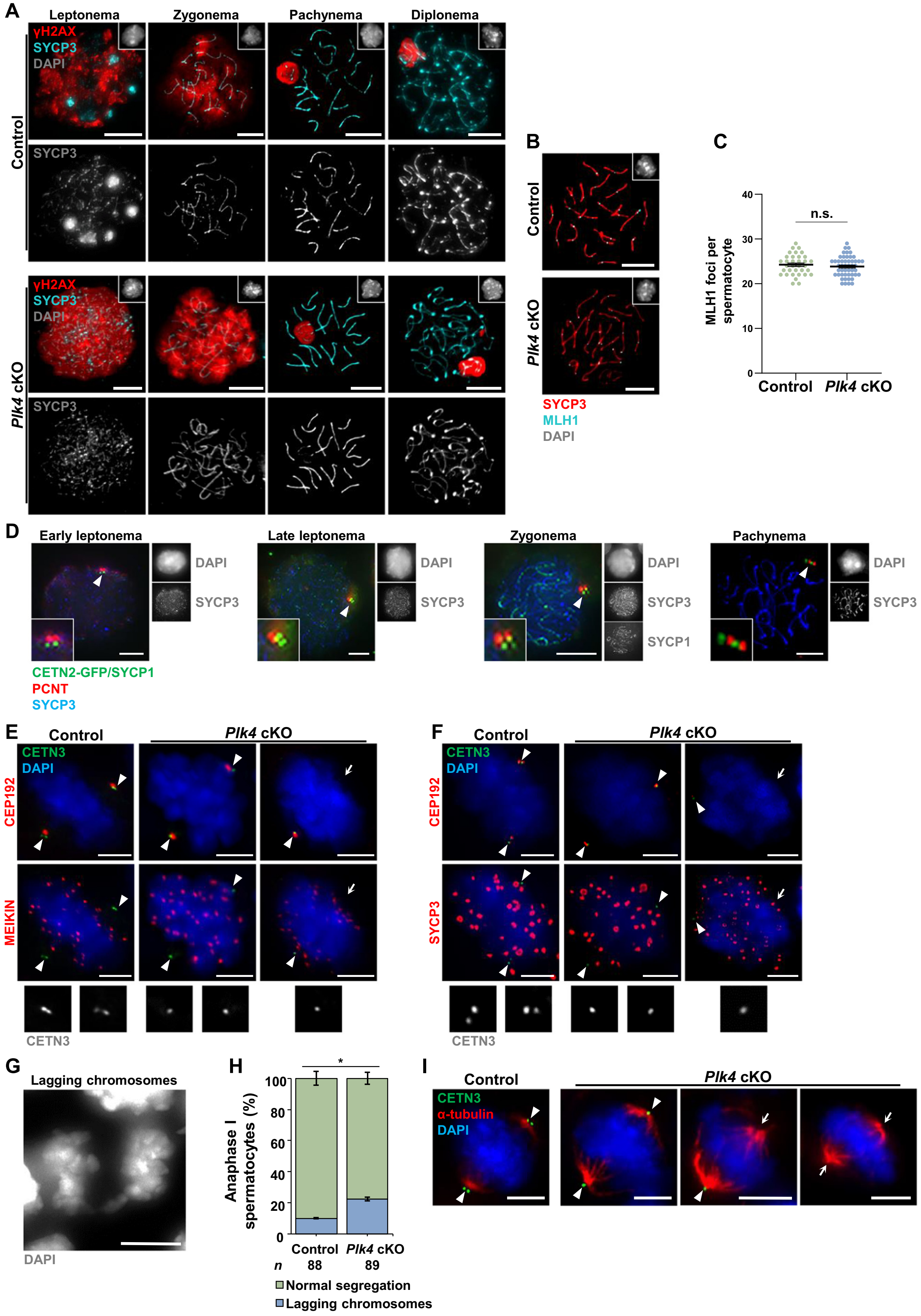

This is a U.S. Government work and not under copyright protection in the US; foreign copyright protection may apply

◀ **Figure EV3. Additional characterization of meiotic progression and centriole biogenesis during prophase I to metaphase II.**

(**A**) Prophase I control and *Plk4* cKO spermatocytes from 14 and 20 dpp mice were immunolabeled against SYCP3 (cyan), γ-tubulin (red), and stained with DAPI (gray inset in the upper right corner). Zoomed images of the centrioles are inset on the corresponding images. Scale bars = 10 µm. (**B**) Representative images of mid-prophase I spermatocytes in 20 dpp control and *Plk4* cKO mice immunolabeled against SYCP3 (red), MLH1 (cyan), and stained with DAPI (gray inset in the upper right corner). Scale bars = 10 µm. (**C**) Quantification of MLH1 foci observed along SYCP3 stretches during pachynema in both control and *Plk4* cKO spermatocytes. Immunolabeling was performed on 3 biological replicates with ≥ 10 spermatocytes quantified per replicate. The total number of cells quantified for control, and *Plk4* cKO mice were 34 and 49, respectively. *P* value = 0.4386. (**D**) Representative images of early leptotene stage, late leptotene stage, zygotene stage, and pachytene stage control spermatocytes expressing CETN2-GFP (green) from 10 dpp mice immunolabeled against SYCP1 (green), PCNT (red), SYCP3 (blue), and stained with DAPI (gray outset). The white arrowheads indicate the centrosome. Individual gray outset channel of SYCP3 and SYCP1 are displayed to the right of the corresponding images. Zoomed images of the centrioles and inset on corresponding images. Scale bars = 5 µm. (**E**) Characterization of metaphase I compared to metaphase II spermatocytes by observing DNA size (stained with DAPI) was confirmed with MEIKIN and SYCP3 immunostaining. Both SYCP3 and MEIKIN staining are present in metaphase I spermatocytes, but no longer in metaphase II spermatocytes (Kim et al, 2015). Representative image of metaphase I control and *Plk4* cKO spermatocytes from 27 dpp mice immunolabeled against CETN3 (green), MEIKIN (red) and stained with DAPI (blue). The white arrowheads indicate the centrosome. The white arrows indicate the ncMTOC. Zoomed images of the centrioles (gray) are displayed below the corresponding images. Scale bars = 5 µm. (**F**) Representative image of metaphase I control and *Plk4* cKO spermatocytes harboring the CETN2-GFP transgene (green) from 27 dpp mice immunolabeled against SYCP3 (red) and stained with DAPI (blue). The white arrowheads indicate the centrosome. The white arrows indicate the ncMTOC. Zoomed images of the centrioles (gray) are displayed below the corresponding images. Scale bars = 5 µm. (**G**) Representative image of chromosome missegregation event during metaphase I stained with DAPI. Scale bars = 5 µm. (**H**) Quantification of lagging chromosomes in anaphase I spermatocytes. Immunolabeling was performed on 5 biological replicates with ≥ 10 spermatocytes quantified per replicate. The total number of cells quantified for control and *Plk4* cKO mice were 89 and 89, respectively. *P* value = 0.0278. (**I**) Representative images of metaphase II spermatocytes in 27 dpp control and *Plk4* cKO mice expressing CETN2-GFP (green) immunolabeled against α-tubulin (red) and stained with DAPI (blue). The white arrowheads indicate the centrosome. The white arrows indicate the ncMTOC. Scale bars = 5 µm. Data information: For all graphs (**C**, **H**), error bars show mean ± SEM. *P* values were obtained from two-tailed Student's *t* test. n.s (not significant), $^*P < 0.05$.

This is a U.S. Government work and not under copyright protection in the US; foreign copyright protection may apply

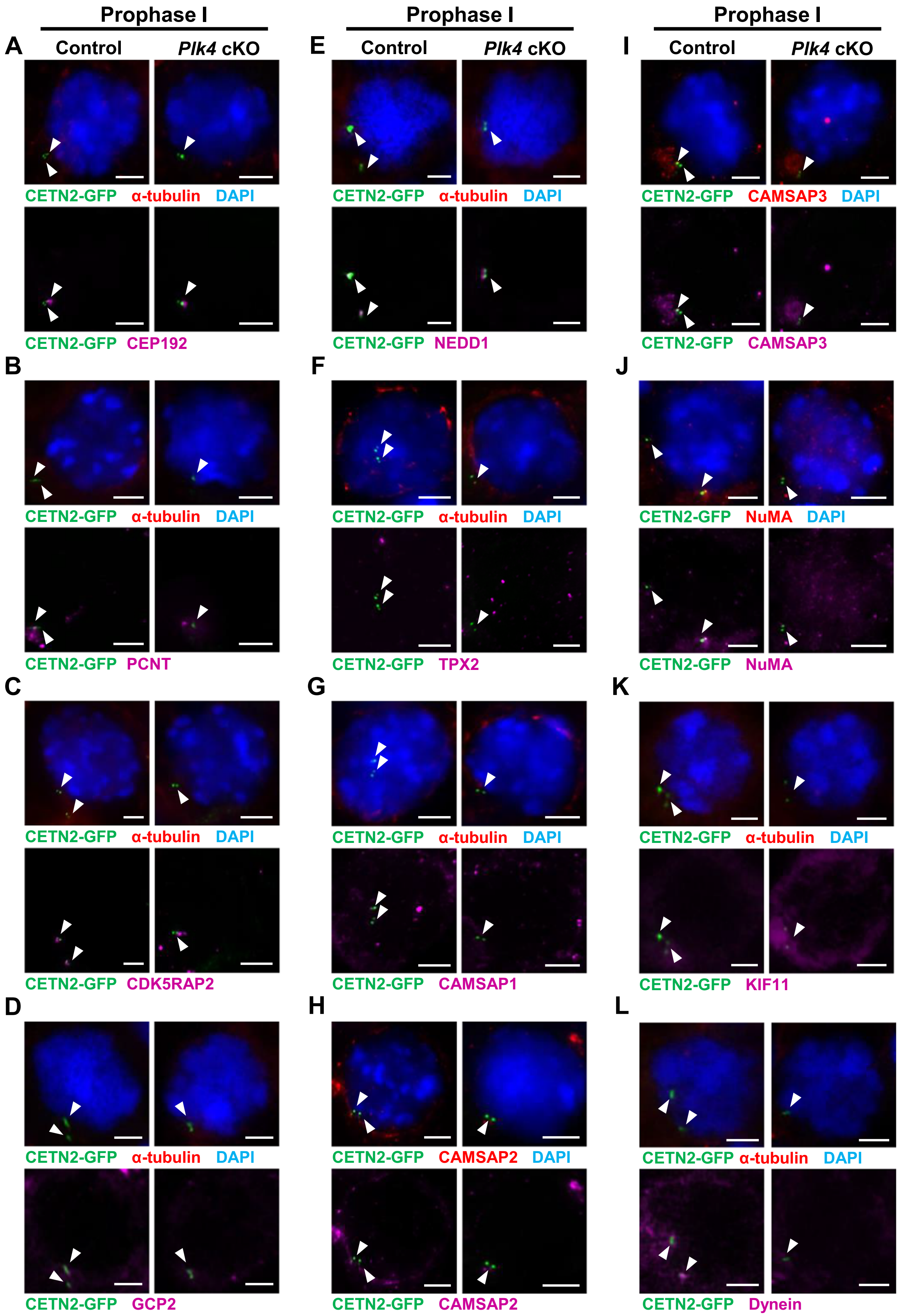

 This is a U.S. Government work and not under copyright protection in the US; foreign copyright protection may apply

Figure EV4. Assessment of microtubule-associated factors during prophase I.

Representative image of prophase I (**A–L**) control and *Plk4* cKO spermatocytes from 24–27 dpp mice expressing CETN2-GFP (green) and stained with DAPI (blue). The white arrowheads indicate the centrosome. The white arrows indicate. Scale bars = 5 µm. (**A**) Immunolabeled against α-tubulin (red) and CEP192 (purple). (**B**) Immunolabeled against α-tubulin (red) and PCNT (purple). (**C**) Immunolabeled against α-tubulin (red) and CDK5RAP2 (purple). (**D**) Immunolabeled against α-tubulin (red) and GCP2 (purple). (**E**) Immunolabeled against α-tubulin (red) and NEDD1 (purple). (**F**) Immunolabeled against α-tubulin (red) and TPX2 (purple). (**G**) Immunolabeled against α-tubulin (red) and CAMSAP1 (purple). (**H**) Immunolabeled against CAMSAP2 (red and purple). (**I**) Immunolabeled against CAMSAP3 (red and purple). (**J**) Immunolabeled against NuMA (red and purple). (**K**) Immunolabeled against α-tubulin (red) and KIF11 (purple). (**L**) Immunolabeled against α-tubulin (red) and dynein (purple).

This is a U.S. Government work and not under copyright protection in the US; foreign copyright protection may apply
